# Experiments indicating a second hydrogen ordered phase of ice VI

**DOI:** 10.1039/c8sc00135a

**Published:** 2018-03-26

**Authors:** Tobias M. Gasser, Alexander V. Thoeny, Lucie J. Plaga, Karsten W. Köster, Martin Etter, Roland Böhmer, Thomas Loerting

**Affiliations:** a Institute of Physical Chemistry , University of Innsbruck , 6020 Innsbruck , Austria . Email: thomas.loerting@uibk.ac.at; b Fakultät Physik , Technische Universität Dortmund , D-44221 Dortmund , Germany; c Deutsches Elektronen-Synchrotron (DESY) , 22607 Hamburg , Germany

## Abstract

In the last twelve years five new ice phases were experimentally prepared. Two of them are empty clathrate hydrates and three of them represent hydrogen ordered counterparts of previously known disordered ice phases. Here, we report on hydrogen ordering in ice VI samples produced by cooling at pressures up to 2.00 GPa. Based on results from calorimetry, dielectric relaxation spectroscopy, Raman spectroscopy, and powder X-ray diffraction the existence of a second hydrogen ordered polymorph related to ice VI is suggested. Powder X-ray data show the oxygen network to be the one of ice VI. For the 1.80 GPa sample the activation energy from dielectric spectroscopy is 45 kJ mol^–1^, which is much larger than for the known hydrogen ordered proxy of ice VI, ice XV. Raman spectroscopy indicates the 1.80 GPa sample to be more ordered than ice XV. It is further distinct from ice XV in that it experiences hydrogen disordering above ≈103 K which is 26 K below the ice XV to ice VI disordering transition. Consequently, below 103 K it is thermodynamically more stable than ice XV, adding a stability region to the phase diagram of water. For the time being we suggest to call this new phase ice β-XV and to relabel it ice XVIII once its crystal structure is known.

## Abstract 2

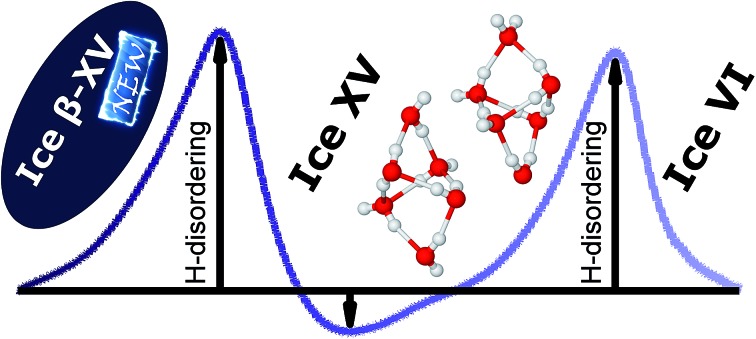
We report on the discovery and characterization of ice β-XV, which represents the second hydrogen ordered polymorph related to ice VI.

## Introduction

One of water's anomalies is its rich polymorphism. Currently 17 crystalline phases are known.^1^ These polymorphs can be categorized as hydrogen ordered and hydrogen disordered, where typically the disordered form has exactly one ordered pendant. Some ordered variants, such as for ice IV, are awaiting their discovery.^2^ Currently no experimentally confirmed examples are known for hydrogen disordered phases with more than one ordered counterpart. This is surprising since many different ways of ordering are possible for a given mother phase.^3^ Ice VI was discovered by Bridgman in the early 20^th^ century, using a piston-cylinder setup allowing him to reach pressures exceeding 1 GPa.^4^ The large tetragonal *P*4_2_/*nmc* unit cell of ice VI contains 10 water molecules. Oxygen atoms are arranged in two interpenetrating but not interconnecting networks of hexamer cages. This “self-clathrate” is one of the most complicated of all known crystalline ice phases.^5^–^7^ There are two crystallographically distinct types of oxygen atoms and four distinct types of hydrogen positions in ice VI.^6^ 45 possible types of ordering were identified.^3^,^6^ In the 1960s partial hydrogen order in ice VI was inferred.^5^ In 1976 Johari and Whalley observed a slow ordering transformation in ice VI at low temperatures.^8^,^9^ More than thirty years later Salzmann *et al.* reported the structure of ice XV, the hydrogen ordered form of ice VI.^10^ This was possible by enhancing the dynamics of hydrogen atoms decorating the oxygen lattice through introducing point defects by HCl doping. While theoretical calculations predicted ferroelectric ordering and *Cc* space group symmetry for ice XV,^11^ experimental data indicate antiferroelectric ordering and *P*1.^6^,^10^ The hydrogen ordering in ice XV does not reach completion, but remains partial. In order to explain why only a partial transformation from ice VI to ice XV is achieved the occurrence of small ferroelectric *Cc* nanocrystallites was proposed, which are destabilized as soon as the dielectric constant of the transforming ice VI is reduced.^12^,^13^ Although ice XV was scrutinized very well by different methods,^3^,^14^–^16^ especially its phase transition behavior is still not fully understood.

**Fig. 1:**
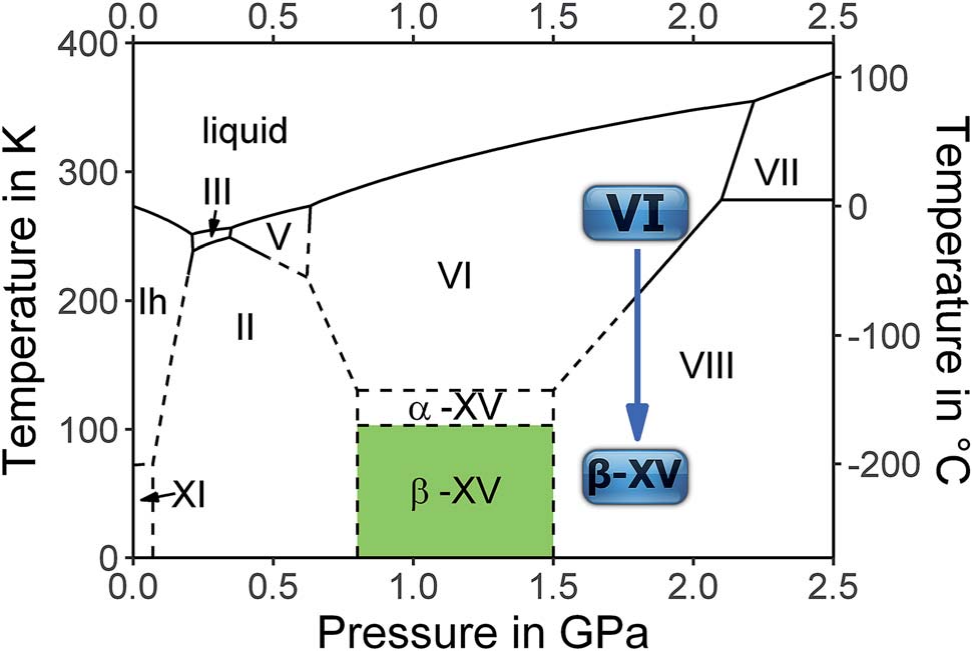
The equilibrium phase diagram of H_2_O including the colored area in which the new hydrogen ordered phase, tentatively called ice β-XV, is stable. The blue arrow at 1.8 GPa shows the cooling process of ice VI from 255 K leading to the new polymorph at 77 K, which is metastable in the ice VIII domain. Dashed lines are extrapolations from experimentally determined phase boundaries (solid lines).

The complicated nature of ice VI involving two unconnected networks implies that point defects migrating in one of them are unable to switch to the other network. This is a source for complex thermal signatures associated with the disordering in ice XV. For other ordered ices, such as ice XI,^17^ ice XIII 18 and ice XIV,^19^ the thermal signatures of hydrogen disordering just involve a single endotherm. For ice XV a weak exotherm preceding the endotherm indicating the first-order transition to ice VI was observed by Shephard and Salzmann upon heating.^15^ This finding was explained by the interaction between the two unconnected networks, where initial ordering in one of them triggers the disordering in the other upon heating.^15^ Similarly, upon cooling intra-network hydrogen ordering takes place at temperatures that are higher than those at which inter-network ordering occurs.^15^ Therefore, a variation of the original preparation protocol of ice XV should affect the ordering scheme of the hydrogen atoms. Specifically, the distance between the two unconnected networks may be altered by changing the pressure, at which ice VI is cooled for the hydrogen atoms to order. We surmise higher pressure to increase the proximity and subsequently the strength of the interaction between the two unconnected networks. Furthermore, we expect the feedback between the two networks to be time-sensitive, such that changes in the cooling rate affect the degree and/or type of hydrogen ordering within ice VI. Based on these hypotheses we studied the influence of preparation pressure and high-pressure cooling rates on the hydrogen ordering. This led us to observations that suggest the existence of a variant of ice VI which is more ordered than ice XV.^10^ The existence of a more ordered phase now adds a rectangular stability region to the phase diagram of ice in the GPa pressure range as shown in Fig. 1.

## Methods

### Preparation of ice samples

The ice XV reference was prepared following the protocol by Shephard and Salzmann,^15^ that is by cooling 600 μl 0.01 M HCl in H_2_O to 77 K, compressing to 1.0 GPa using our ZWICK BZ100 material testing machine with a piston-cylinder setup (8 mm diameter bore), heating to 255 K and quenching with liquid nitrogen at ≈–45 K min^–1^. The ice VI reference was prepared the same way except that no dopant was introduced, *i.e.*, pure H_2_O instead of 0.01 M HCl was used. The new polymorph identified in this work was prepared by varying this protocol. After cooling 0.01 M HCl to 77 K at 1 bar, pressures of 1.0–2.0 GPa were applied before heating to 255 K. To induce hydrogen ordering the subsequent high-pressure cooling rate was reduced to ≈3 K min^–1^. At 77 K the pressure is released to recover the hydrogen ordered ice at ambient pressure.

Except for the ice VI and ice XV references, all samples studied in this work are cooled at ≈3 K min^–1^. Therefore, we will refer to those samples based on their preparation pressure. That is, in the following the term “1.80 GPa sample” refers to a sample prepared by cooling ice VI from 255 to 77 K at 1.80 GPa and ≈3 K min^–1^. In addition to studying the quench-recovered ices directly, we also studied these latter ice samples after heating and recooling them at ambient pressure. This procedure was described by Whale *et al.* in the literature.^16^ Here such samples are referred to as “recooled ices”, *e.g.*, in the following the term ice XV_rec(130 K)_ will refer to a recooled ice XV reference sample that is studied at ambient pressure after heating to 130 K and recooling to 77 K. A 1.80 GPa_rec(114 K)_ sample refers to a sample of the new polymorph that was heated to 114 K and then recooled at ambient pressure.

### Differential scanning calorimetry

All ices were scrutinized calorimetrically using a Perkin Elmer DSC 8000 and compared with earlier work of Shephard and Salzmann.^15^ The samples were filled into aluminum crucibles under liquid nitrogen and directly transferred to the precooled oven of the calorimeter. Every sample was characterized by heating at 10 K min^–1^ from 93 to 253 K – which first results in the disordering of the H-atom network and subsequently to a rearrangement of the O-atom network to produce ice I.^15^,^20^ To determine the sample mass used for the measurement, the endothermic melting peak at 273 K was compared with the known heat of fusion of water, 6012 J mol^–1^.^21^ Similar to the method used by Shepard and Salzmann^15^ the baseline was interpolated between the straight sections of the baseline before the first and after the second endotherm to evaluate the peak areas. The reproducibility of the enthalpies associated with the endotherm is ±7 J mol^–1^, independent from the baseline choice and including all random and systematic errors. Additionally recooling experiments^15^,^16^ at 117 K were conducted to probe the reversibility of the (dis)ordering transition at ambient pressure by keeping the DSC oven at 117 K for 30–60 minutes, recooling to 93 K and heating at 10 K min^–1^.

### Dielectric relaxation experiments

To measure the dielectric response of the ice crystals, the samples were transferred from Innsbruck to Dortmund under liquid nitrogen conditions. A Novocontrol Alpha-A impedance analyzer was used to investigate the dielectric response as described in earlier works.^19^,^22^ The powdered crystals were cold-loaded into a parallel plate capacitor connected to a Quatro cryosystem. The dielectric losses are presented in arbitrary units, since an exact determination of the cell's filling factor was not possible. Dielectric loss spectra were obtained for the 1.80 GPa sample upon heating from 96 K. These measurements were carried out for three samples taken from the same batch. Thereby, we checked whether the behavior that we associate with the existence of ice β-XV is reproducible. Using average rates of 0.1 to 0.6 K min^–1^ the 1.80 GPa sample was heated directly to the temperature range above 130 K, in which ice VI is stable. In addition we studied sample 1.80 GPa_rec(114 K)_ and heated it from 102 to above 130 K. Finally, an ice XV reference sample prepared at 1.00 GPa was also measured. In the Arrhenius plots that will be used to determine activation energies, closed symbols represent relaxation times, which were evaluated by fitting the dielectric loss peak using a Havriliak–Negami function;^23^ open symbols were assessed by frequency-temperature-superposition.^19^,^22^

### Raman spectroscopy

All Raman spectra were recorded with a WiTec WMT50 Spectrometer (532 nm laser, 20 mW) inside an Oxford N Microstat controlled by a Lakeshore 331S temperature controller. Samples were powdered and loaded into the microstat at 84 K. Crystals were selected under the microscope for Raman measurements in confocal geometry. Raman spectra were taken using accumulation times of about 30 minutes. The data are smoothed with a 2^nd^-order Savitzky–Golay filter (7 points). Samples containing 5 wt% D_2_O were studied in order to decouple the OD from the OH vibrations as in many studies before.^16^,^24^,^25^ We made crosschecks whether the ordering is possible in H_2_O samples containing 5 wt% D_2_O by comparing these samples with pure H_2_O samples. The agreement of DSC scans and OH and translational regions in Raman spectra shows that the ordering to the new polymorph is not affected by adding small amounts of D_2_O.

### Powder X-ray diffraction

For powder X-ray diffraction measurements samples were powdered under liquid nitrogen and transferred to the sample chamber. X-ray patterns were recorded in flat geometry on a Cu sample holder with an XPERT III PANalytical Xpert Pro MPD diffractometer (Fig. 10(a), sample loaded at 20 K) or on a Cu–Ni sample holder with a Siemens D5000 diffractometer (Fig. 10(b) and (c), sample loaded at 80 K). The peak intensities were scaled to match at 0.265 nm. All diffractograms were obtained using a Cu Kα X-ray source (1.5406 Å) in θ–θ geometry and using a Göbel-mirror for parallel-beam optics. The instruments are calibrated regularly by measuring a quartz reference.

## Results

### Calorimetry experiments

#### Pressure and cooling rate dependence

**Fig. 2:**
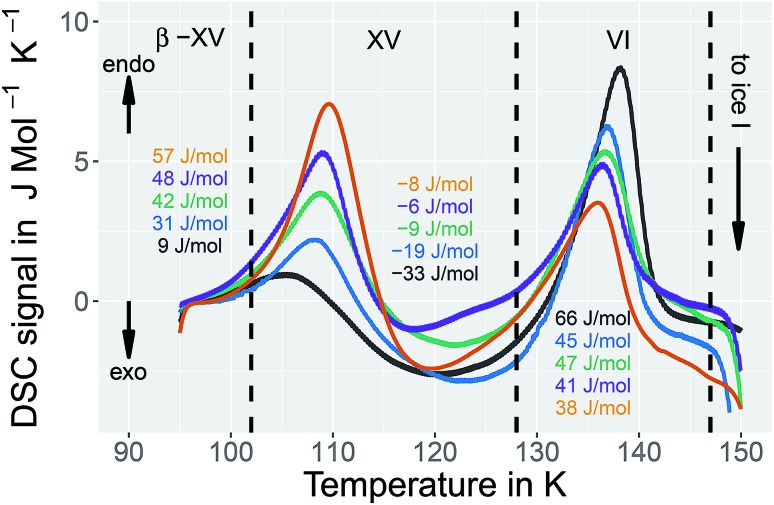
Differential scanning calorimetry (DSC) thermograms of samples of the new polymorph prepared at pressures of 1.00 GPa (black), 1.45 GPa (blue), 1.60 GPa (green), 1.80 GPa (violet) and 2.00 GPa (orange) and cooled at the given pressures with a rate of approximately 3 K min^–1^. The baselines were corrected and shifted to 0 J mol^–1^ K^–1^ at 96 K. The indicated enthalpies are average values from 2 (orange), 19 (violet), 4 (green), 5 (blue) and 8 (black) scans. Heating rate is 10 K min^–1^ for all DSC traces.

Previously, ice XV was prepared by quenching HCl-doped ice VI kept at 1.00 GPa at rates of about 45 K min^–1^.^15^ Calorimetry studies carried out by heating ice XV from 100 K at ambient pressure have revealed a weak exotherm preceding an endotherm with an onset at 129 K.^15^ This endotherm was shown to indicate the hydrogen disordering transition transforming ice XV to ice VI. By reducing the cooling rate at 1.00 GPa to 3 K min^–1^ a weak endotherm of 9 J mol^–1^ appears in our calorimetry experiments at 1 bar at an onset temperature of 103 K (black trace in Fig. 2), that was not noticed for samples cooled at 45 K min^–1^.^15^ By increasing the pressure to 1.45, 1.60, 1.80 and 2.00 GPa this previously unknown endotherm grows to 31, 42, 48 and 57 J mol^–1^, respectively. On the other hand, in the same pressure range the endotherm with an onset at 129 K indicating the ice XV → ice VI disordering transition shrinks from 66 to 38 J mol^–1^ (see Fig. 2). For 1.80 and 2.00 GPa the size of the first endotherm discovered in this work exceeds the size of the previously known second one (orange and violet traces in Fig. 2). Furthermore, the increase in preparation pressure shifts the ice XV → ice VI endotherm down by 2 K, but the new endotherm shifts up by 4 K. The data in Fig. 2 represent an observation of two well-separated endotherms. As we will show below, both endotherms are associated with hydrogen disordering – based on powder X-ray diffraction the oxygen network found for ice XV as well as for ice VI is also present for samples cooled at 1.80 GPa. The exotherm noted in ref. 15 is analogously seen in Fig. 2 between the two endotherms, but it is comparably weak. We think the exotherm reflects enthalpy relaxation following the reordering of H atoms after the first endotherm. The configurational entropy changes by 0.55 J K^–1^ mol^–1^ at the first peak and 0.30 J K^–1^ mol^–1^ at the second for the 2.0 GPa sample. This corresponds to 16% and 9% of the Pauling entropy, the latter referring to the maximum entropy change achievable through disordering hydrogen atoms in ice samples.

### Recooling experiments at 1 bar

In order to learn more about the nature of the 1.80 GPa sample we have conducted recooling experiments at 1 bar. It is well known that recooling of HCl doped ice VI from 135 K to 77 K at 1 bar results in ice XV.^15^ In DSC scans this means that upon cooling from 135 K an exotherm appears that is of the same size as the endotherm at 129 K. In a second heating run the endotherm at 129 K reappears. In other words, it is possible to cycle between ice VI (above 129 K) and ice XV (below 129 K) at ambient pressure.

**Fig. 3:**
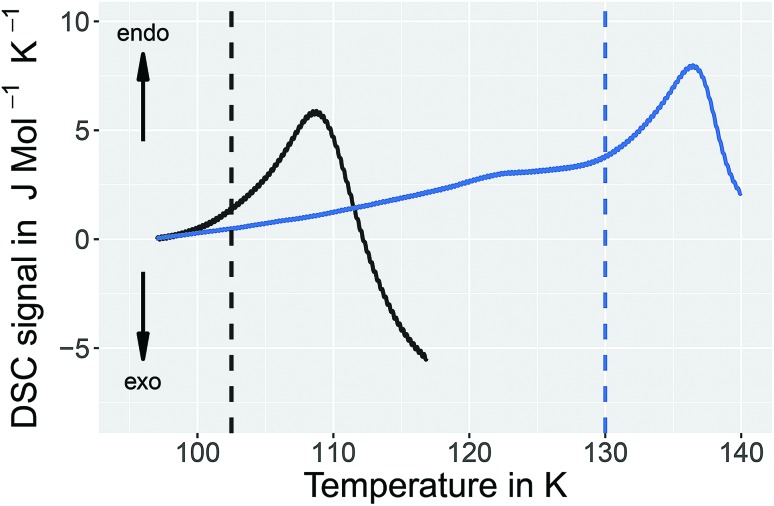
Influence of recooling at ambient pressure on the 1.80 GPa sample (ice β-XV). DSC thermograms of samples prepared at 1.80 GPa heated to 117 K (black trace), recooled to 93 K at 1 K min^–1^ and heated a second time to 140 K (blue trace). Heating rate is 10 K min^–1^ for all DSC traces. The baselines were corrected and shifted to 0 J mol^–1^ K^–1^ at 97 K.

It is, however, not possible to cycle the previously unknown phase at 1 bar as shown in Fig. 3 for the 1.80 GPa sample. On first heating at 1 bar the first endotherm appears just like in Fig. 2, see Fig. 3 black trace. When subsequently recooling the 1.80 GPa sample from 117 K, a temperature that was chosen to avoid the transformation to ice VI, an exotherm does not appear at 1 bar (not shown in Fig. 3). Neither did we observe the first endotherm at 103 K in the second heating run (see Fig. 3, blue trace). Except for a little kink at about 123 K which marks the beginning of the remaining part of the exotherm, a flat baseline is seen upon second heating. This (i) confirms that the endothermic feature is not a glass transition (which has to reappear in cooling–heating cycles) and (ii) implies that the previously inaccessible phase does not form upon recooling at 1 bar. The ice XV → ice VI endotherm, however, appears for the second heating in the blue trace of Fig. 3. It is located at the same temperature as in Fig. 2, suggesting that it indicates the ice XV to ice VI transition. This endotherm reappears at the same temperature also in a third heating after recooling from ice VI at 130 K (not shown in Fig. 3). This finding is consistent with the idea that the second endotherm indeed corresponds to the hydrogen disordering from ice XV to ice VI. That is, the previously inaccessible polymorph, to be characterized further below, transforms to ice XV/VI above 103 K at 1 bar. It does not form at 1 bar from ice XV/VI, and it also does not form at 1.00 GPa from ice VI. It only forms at higher pressures, *e.g.*, at 1.80 GPa, on slow cooling of ice VI. Thus, the new endotherm appears to indicate a phase transition between two phases.

This naturally raises the question what the two phases before and after the first endotherm are. Our temperature dependent X-ray studies (see below) indicate that the network of oxygen atoms is the same as the one in ice VI. At ambient pressure this network is retained up to about 150 K, where the oxygen network rearranges to form ice I. An amorphous nature and all other known ice polymorphs, *i.e.*, ice I–V, ice VII–XIV, ice XVI and ice XVII can be excluded on the basis of the X-ray results. The only phases to be considered are ice VI, ice XV and all other possible hydrogen ordered phases based on the ice VI oxygen network. Under these premises two interpretations can be invoked to understand the DSC scans from the 1.80 GPa sample, both of which involve a previously experimentally inaccessible hydrogen ordered phase related to ice VI. The first interpretation involves a heterogeneous sample containing domains of ice XV and the previously inaccessible ordered phase. Upon disordering both domains convert to ice VI, one at 103 K, the other (ice XV domains) at 129 K. The second interpretation involves a homogeneously ordered sample that converts to ice VI in two distinct steps, where both steps involve hydrogen disordering. In that latter interpretation the previously inaccessible phase transforms in the first step at 103 K to a less hydrogen ordered phase, which is presumably ice XV, which in the second step at 129 K disorders to produce ice VI.

### Dielectric experiments

In order to learn more about the nature and the relaxation dynamics of the 1.80 GPa sample we have studied quench-recovered crystals using dielectric spectroscopy. Runs on three different 1.80 GPa samples obtained from the same batch were performed. During the first two runs dielectric loss spectra were recorded by heating the sample to temperatures at which ice VI is stable. As part of the third run the 1.80 GPa sample was recooled after heating it up to 114 K resulting in the 1.80 GPa_rec(114 K)_ sample. For comparison, we have also studied an ice XV reference sample prepared at 1.00 GPa. Based on the previous section for this reference sample an additional phase transition (related to the first endotherm at 103 K) is not expected.

**Fig. 4:**
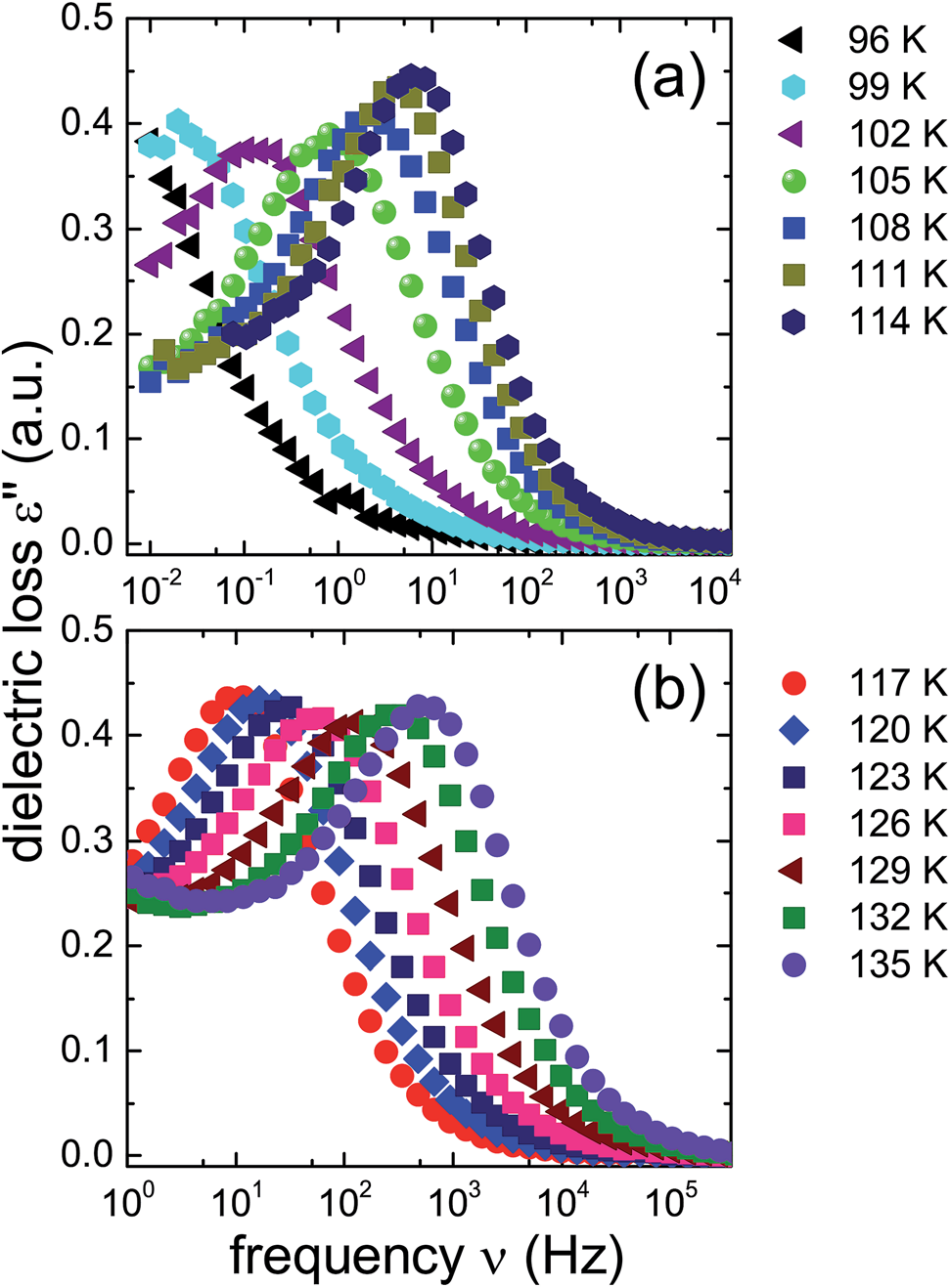
Dielectric loss spectra of the 1.80 GPa sample (ice β-XV) during first heating. Spectra acquired for *T* ≤ 114 K are shown in panel (a) and data referring to higher temperatures are shown in panel (b). Please note that the frequency axes of the two panels are different.

Dielectric loss spectra were obtained upon heating in temperature steps of typically Δ*T* = 3 K. Fig. 4(a) depicts dielectric loss spectra recorded for the 1.80 GPa sample in the temperature range from 96 to 114 K and Fig. 4(b) covers the range from 117 to 135 K. For increasing temperatures the spectra shift to higher frequencies which *via τ* = 1/(2π*ν*_peak_) signals a reduction of the dipolar relaxation times *τ*. An interesting feature is observed in Fig. 4(a) when comparing the spectra recorded below with those recorded above 105 K: the shift of the spectra along the frequency axis induced by each Δ*T* step changes significantly near 105 K. This behavior was detected in all runs carried out for the three different 1.8 GPa samples (see below). The dielectric data presented in this work thus hint at the occurrence of a phase transition near 105 K, in accord with the conclusion drawn from the first, low-temperature endotherm observed in DSC scans.

**Fig. 5:**
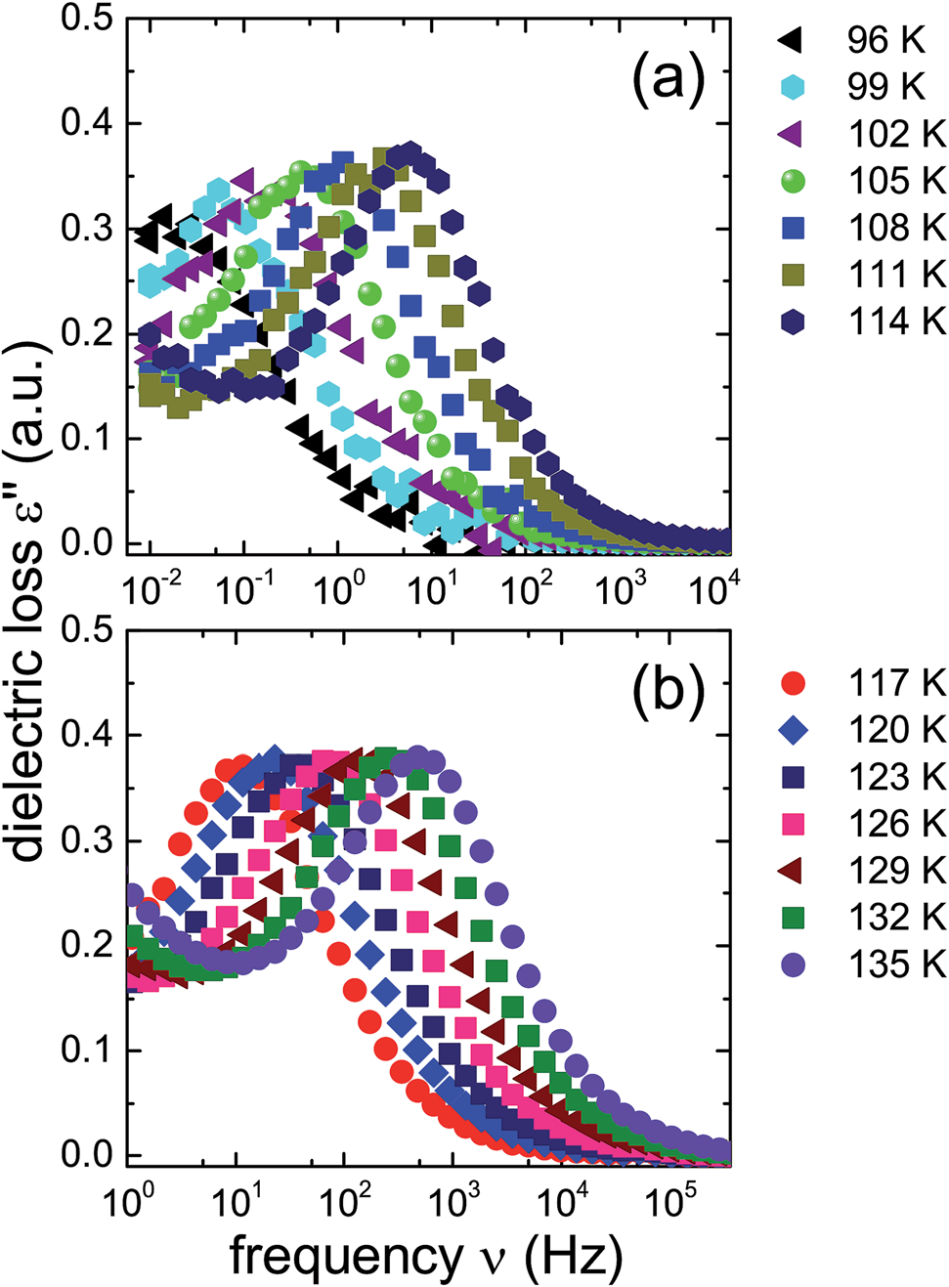
Dielectric loss spectra of the reference ice XV sample produced at 1.00 GPa, recorded upon heating. As in Fig. 4 spectra for *T* ≤ 114 K are shown in panel (a) and those for *T* ≥ 114 K are displayed in panel (b). Please note that the frequency axes of the two panels are different.

The ice XV reference sample was studied in the same temperature range and the corresponding dielectric loss spectra are shown in Fig. 5. Clear-cut changes of temperature-induced shifts between adjacent spectra are not discernible from these data, in particular in the temperature range from 96 to 114 K (Fig. 5(a)) as well as that from 117 to 135 K (Fig. 5(b)). Thus, the temperature induced shifts of the dielectric loss spectra of the reference sample do not yield indications for the occurrence of a low-temperature phase transition, different from what is observed for the 1.80 GPa sample.

The new polymorph prepared at 1.80 GPa and the ice XV reference sample prepared at 1.00 GPa differ not only regarding the temperature evolution of the relaxation times but also with respect to the amplitudes of the dielectric losses, in particular when focusing on the temperature range from 117 to 135 K: while in Fig. 5(b) the peak amplitude is temperature independent for the reference sample, the amplitude for the 1.80 GPa sample decreases with increasing temperature for *T* < 129 K followed by an increase in amplitude (Fig. 4(b)). Thus, while the relaxation time measurements do barely reveal the existence of the phase transition to ice VI, the amplitude measurement does. It is unclear at present, why the ice XV → ice VI transition is not well resolved for the reference sample, a situation that is resembling the one for the ice XI → ice I transition in KOD doped ice.^26^ By contrast, the disordering transition of the 1.80 GPa sample is clearly seen in terms of amplitudes decreasing below, but increasing above 102 K in Fig. 4(a).

**Fig. 6:**
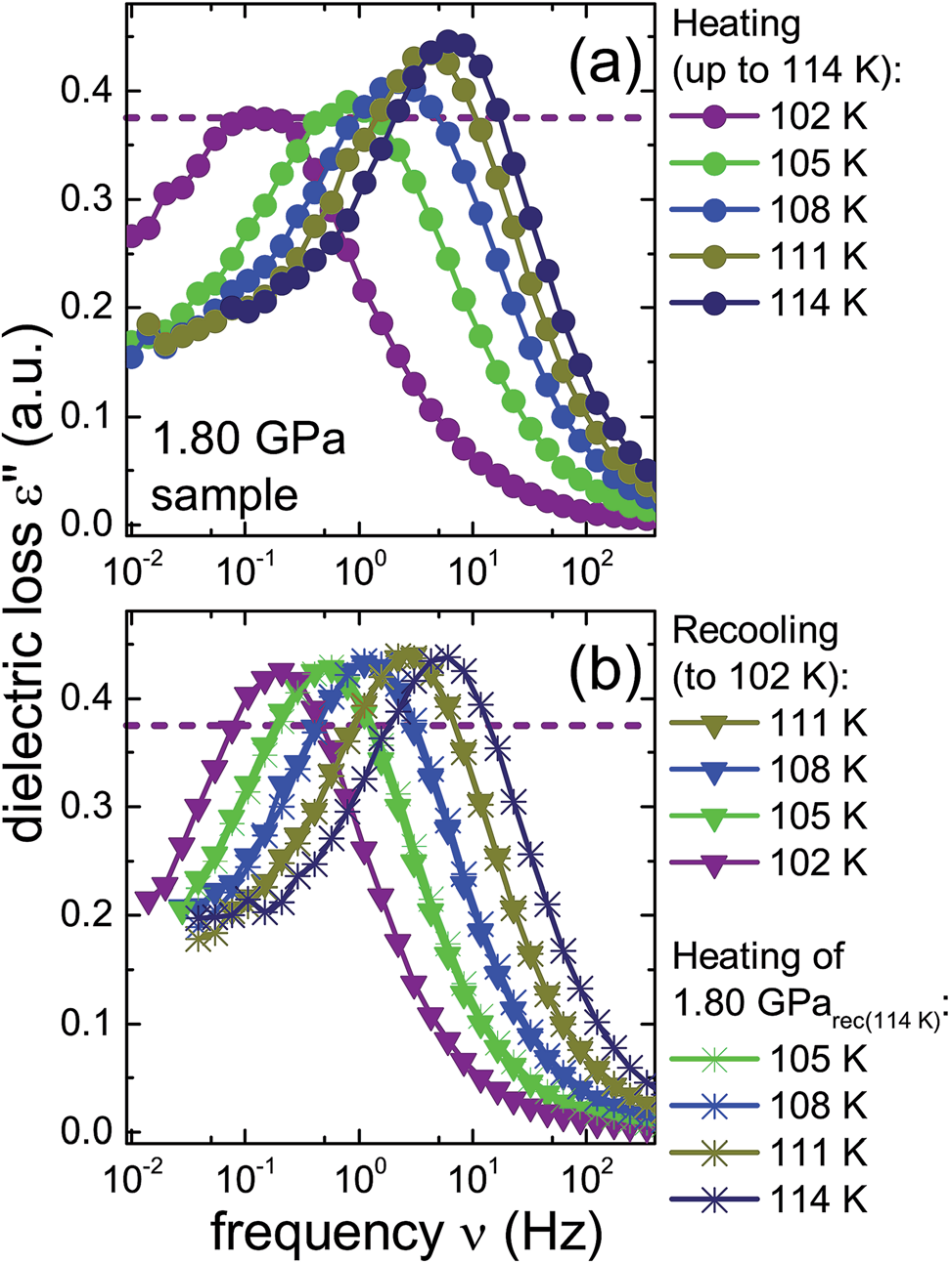
Dielectric loss spectra of the same 1.80 GPa sample (ice β-XV) for which data are shown in Fig. 4. (a) Spectra measured upon first heating to 114 K are re-plotted here with a zoomed frequency scale to facilitate comparison with subsequent measurements on this sample. (b) Triangles represent data obtained during subsequent cooling which yielded sample 1.80 GPa_rec(114 K)_. The spectra recorded upon heating of this sample are represented in (b) as asterisks. The dashed lines refer to the amplitude of the dielectric loss spectrum measured at 102 K for the 1.80 GPa sample upon first heating. To guide the eye, data points are connected by lines.

To further investigate the nature of the previously inaccessible low-temperature state, Fig. 6 compares the dielectric loss spectra of the 1.80 GPa sample, Fig. 6(a), with those for the 1.80 GPa_rec(114 K)_ sample, Fig. 6(b), that were taken subsequent to heating the sample to 114 K. After recooling and subsequent reheating a reproducible behavior is revealed. This behavior differs, however, from that obtained during the first heating of the 1.80 GPa sample, *cf.*Fig. 6(a). These deviations concern the peak positions as well as the amplitudes of the spectra and indicate that under ambient pressure the transition to the possible low-temperature state is not reversible.

A comparison of the spectra of the 1.80 GPa samples recorded at 102 K with and without prior recooling reveals that the spectrum of the 1.80 GPa_rec(114 K)_ sample is narrower, *cf.*Fig. 6. This observation suggests that below 105 K the 1.80 GPa sample is inhomogeneous in nature. Such an inhomogeneity may arise either in the presence of a mixture of phases or of internal stresses that modulate the barriers against dipolar reorientation. Going back to the two possible interpretations suggested above from the calorimetry data in Fig. 2 and 3, this finding does not support the assumption of a homogeneously ordered sample and favors the presence of distinct domains, that is ice XV domains and domains of a differently ordered variant of ice VI.

**Fig. 7:**
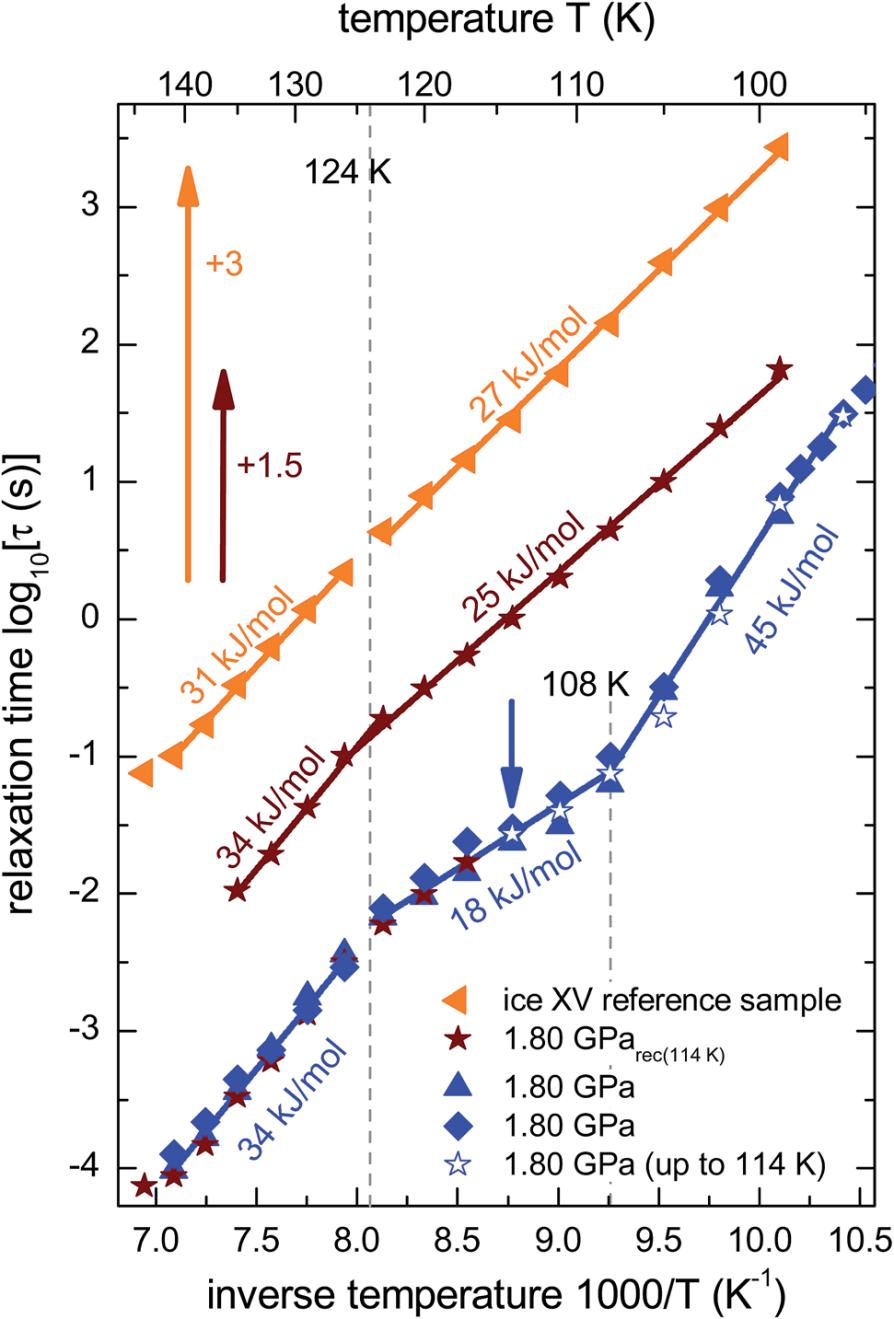
Arrhenius diagram reflecting data recorded upon heating of (from top to bottom) the reference XV sample (*cf.*Fig. 5b), sample 1.80 GPa_rec(114 K)_ (*cf.*Fig. 6b) and the 1.80 GPa sample (ice β-XV). To enhance the clarity of presentation, the relaxation times for sample 1.80 GPa_rec(114 K)_ are shifted up by 1.5 decades and those for the reference ice XV sample by 3 decades. The region of the low-temperature phase transition of the 1.80 GPa sample was examined in three independent measurements (blue symbols). The open stars correspond to the first heating run of the 1.80 GPa sample (*cf.*Fig. 6a) up to 114 K (indicated by the blue arrow), whereas the closed stars refer to the heating of the 1.80 GPa_rec(114 K)_ sample (second heating, *cf.*Fig. 6b). Dotted lines indicate phase transition temperatures for the 1.80 GPa sample. The solid lines correspond to Arrhenius laws with the activation energies indicated in the figure. The uncertainty of the various activation energies is estimated to be about ±2 kJ mol^–1^.

From the frequencies *ν*_peak_ at which the dielectric loss peaks appear, the temperature dependent relaxation times *τ* can be obtained. Fig. 7 summarizes the relaxation maps obtained for the 1.80 GPa samples with and without prior recooling as well as for the ice XV reference sample prepared at 1.00 GPa. Upon heating, the 1.80 GPa sample displays three linear regimes with kinks near 108 K and near 124 K which we associate with the occurrence of phase transitions. From the Arrhenius law
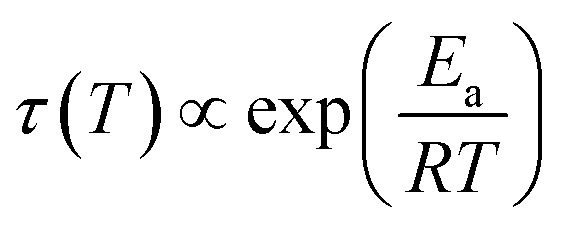
activation energies *E*_a_ are calculated for each linear regime. For the 1.80 GPa sample this results in an activation energy of 45 kJ mol^–1^ below 108 K, an activation energy of 18 kJ mol^–1^ from 108 to 124 K, and 34 kJ mol^–1^ above this temperature. Dotted lines in Fig. 7 mark the intersection of the linear fits which we interpret as phase transition temperatures. These findings are qualitatively consistent with the observation of the two endotherms in Fig. 2. The two temperatures, at which the phase transitions appear are very similar to the ones inferred from the calorigrams in Fig. 2. That is, also dielectric relaxation spectroscopy suggests the presence of three distinct phases, only two of which (ice VI and ice XV) are known from previous experiments.

The relaxation map of the recooled 1.80 GPa_rec(114 K)_ sample displays only one kink near 124 K, see Fig. 7. This finding confirms the above indication that at ambient pressure the transition taking place near 108 K in the pristine sample is not reversible. That is, the new low-temperature phase has disappeared at ambient pressure after prior recooling, again in agreement with the findings from calorimetry. The ice XV reference sample does not show clear signs for a phase transition directly in the dielectric loss spectra. However, *via* a separate analysis of the dipolar relaxation times above and below the phase transition temperatures observed in the 1.80 GPa sample, a slight change of the activation energy of the ice XV to ice VI transition becomes visible. In the intermediate temperature regime the activation energy of the 1.80 GPa sample differs from that of sample 1.80 GPa_rec(114 K)_ and from that of the ice XV reference sample. This behavior cannot be explained with our current knowledge, but might be due to different degrees of relaxation in the H-network for samples that were or were not previously recooled at ambient pressure.

**Fig. 8:**
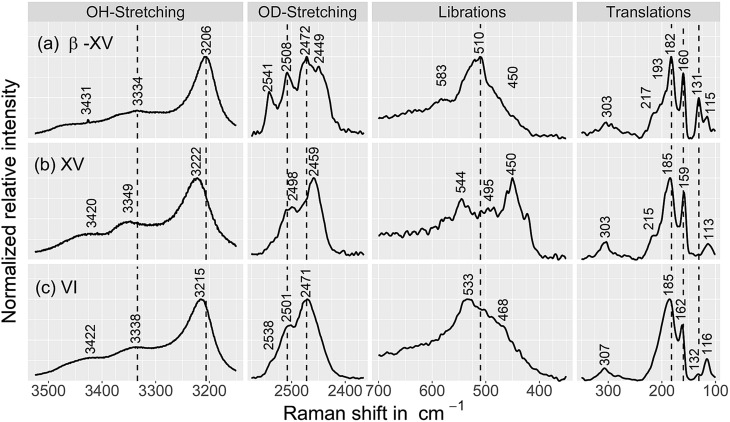
Raman spectra of (a) the 1.80 GPa sample, tentatively designated ice β-XV as well as of (b) ice XV_rec(130 K)_ and (c) ice VI samples, containing 9 mol% HDO. The ice XV sample was recooled from 130 K in the microstat at 10 mbar as described by Whale *et al.*^16^ All spectra were recorded at 84 K. Spectra were smoothed by a 7-point Savitzky–Golay filter and normalized to the most intense peak for each individual trace.

Taken together, the dielectric data hint at the existence of a low-temperature phase transition in the 1.80 GPa sample that occurs in addition to that from ice XV to ice VI. The new low-temperature phase (i) appears to be inhomogeneous, probably due to the presence of a mixture with ice XV or ice VI and (ii) undergoes a phase transition for temperatures near 108 K which under ambient pressure is non-reversible.

### Raman spectroscopy

#### The 1.80 GPa sample compared with ice VI and ice XV

**Fig. 9:**
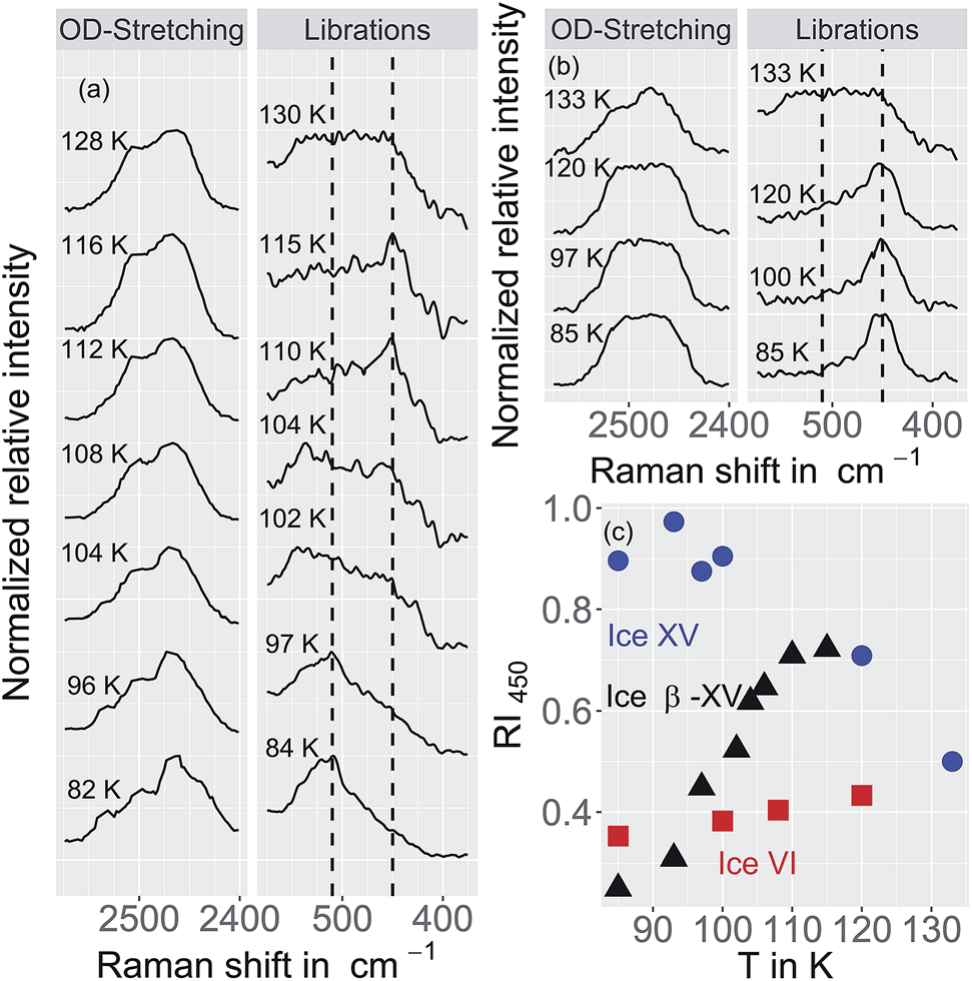
(a) Raman spectra of ice β-XV acquired upon heating. (b) Raman spectra of ice XV (recooled from 120 K at 1 bar) at different temperatures. Spectra are normalized to the most intense peak in each individual trace. A Savitzky–Golay filter was not applied for the processing of these spectra. (c) Relative intensity RI_450_ of the librational band at 450 cm^–1^ (see text for definition). Error bars are on the order of the size of the symbols.

The nature of the hydrogen ordering was additionally scrutinized using Raman spectroscopy (Fig. 8 and 9) because this method is sensitive to the local molecular environment and well suited to discriminate between differently hydrogen ordered phases. In particular, hydrogen ordering leads to a narrowing and/or splitting of bands.^27^ This was shown for instance for the ice XIII/V pair, which features 3–4 separated translational peaks and shoulders at 100 K in the ordered ice XIII phase, but only a single broad peak for disordered ice V at 110 K.^24^Fig. 8 compares the 1.80 GPa sample (panel a), with the ice XV_rec(130 K)_ reference (panel b) and ice VI reference (panel c). Please note that the ice XV in panel b was recooled from 130 K at ambient pressure. The comparison between the spectra for these three samples (all recorded at 84 K) is quite revealing. In general, the 1.80 GPa sample shows the most narrow and most structured bands, see Fig. 8. In the OD stretching region four band maxima are resolved, compared to two in ice VI and ice XV. In the translational regime the 1.80 GPa sample features the sharpest bands among all samples. There are significant shifts in peak positions between ice XV and the 1.80 GPa sample in the OH/OD stretching regions, *e.g.*, the bands at 3206 cm^–1^ and 2449 cm^–1^ are blueshifted by 16 and 10 cm^–1^ compared to ice XV, respectively. Similar effects, including band shifts to higher and lower wavenumbers upon ordering, were observed for ice XII and XIV.^24^,^28^ Furthermore the peak intensities change significantly for selected bands, *e.g.*, the band at 131 cm^–1^ is absent for ice XV (panel b) but of medium intensity for the 1.80 GPa sample (panel a). The three librational bands near 450 cm^–1^, 500 cm^–1^ and 540 cm^–1^ are strong, weak and strong for ice XV, but absent, strong and absent for the 1.80 GPa sample. These observations indicate that the 1.80 GPa sample is more ordered than ice XV.

### Spectral evolution upon heating

Fig. 9 compares the librational and OD-spectra of the 1.80 GPa sample upon heating (panel a) with the ones for a recooled ice XV sample (panel b). Specifically, the ice XV reference was recooled from 120 to 85 K before recording the spectra shown in Fig. 9(b). The first finding to notice is that the Raman spectra for ice XV_rec(130 K)_ (Fig. 8, middle) and ice XV_rec(120 K)_ (Fig. 9(b)) are different. Specifically, the librational band at 544 cm^–1^ is absent for ice XV_rec(120 K)_ and the intensity ratio for the two OD-stretching band differs significantly. This implies that there is a difference in terms of the extent of hydrogen ordering in ice XV prepared by recooling at 1 bar from different temperatures. This issue will be studied and discussed further in a forthcoming publication. Upon heating ice XV_rec(120 K)_ from 85 to 120 K, *i.e.*, just prior to the transition to ice VI, there are barely any spectral changes (see Fig. 9(b)), *i.e.*, ice XV_rec(120 K)_ remains stable up to 120 K. The completed transition to ice VI is evident for the spectra recorded at 133 K. The OD-band shape has changed and adopts the intensity ratios also seen for the reference spectrum of ice VI in Fig. 8 (bottom). Similarly, the transition to ice VI is evident by the growth of the librational band near 533 cm^–1^. By contrast, upon heating the 1.80 GPa sample the spectra change significantly prior to reaching 120 K. While four maxima in the OD-stretching part are resolved at 82 K, only two maxima are resolved at 108, 112, 116 and 128 K. At 96 K and 104 K an intermediate situation is encountered. In the librational regime the most intense band is the 510 cm^–1^ band up to 97 K, whereas the 450 cm^–1^ band is the most intense one at 110 K. At 102 and 104 K the 450 cm^–1^ band is seen to grow, whereas the band at 510 cm^–1^ redshifts to 533 cm^–1^. This indicates that the librational bands are particularly sensitive to hydrogen ordering and useful to investigate phase changes in the H-atom network. Clearly, the changes near 104 K are associated with the first endotherm in Fig. 2 and the change in activation energy in Fig. 7. The pattern changes near 104 K and comparison with the spectra in Fig. 8 favor an interpretation in terms of a hydrogen disordering transition at 104 K. Based on these spectra it is not straightforward to answer whether or not the hydrogen order develops towards that in ice VI or towards that in ice XV at the transition. The growth of the feature at 450 cm^–1^ suggests ice XV formation from the 1.80 GPa sample, whereas the growth of the feature at 533 cm^–1^ suggests ice VI formation. The band at 533 cm^–1^ shows a decreased intensity again at 110 and 115 K in Fig. 9(a), different from the ice VI reference in Fig. 8 (bottom).

Since the librational peaks at 450 cm^–1^, 510 cm^–1^ and 533 cm^–1^ are overlapping, we have analyzed peak heights rather than the peak area. In particular we have analyzed the heights at 450 cm^–1^ and 510 cm^–1^ and defined the relative intensity of the band at 450 cm^–1^ RI_450_ as follows: RI_450_ = *I*_450_/(*I*_450_ + *I*_510_), where *I*_450_ and *I*_510_ denote the intensities at 450 cm^–1^ and 510 cm^–1^, respectively. Fig. 9(c) shows RI_450_ as deduced from the data in Fig. 9(a) and (b) while heating the 1.80 GPa sample (black triangles), ice XV_rec(120 K)_ (blue circles) and ice VI (red squares, spectra not shown). For ice VI the intensity ratio RI_450_ slowly increases from 0.4 at 100 K to 0.5 at 132 K. For XV_rec(120 K)_ the RI_450_ ratio is 0.90 ± 0.05 below 100 K and decreases to 0.7 at 120 K and then to 0.5 at 132 K as the sample transforms to ice VI. For the 1.80 GPa sample RI_450_ is 0.28 at 84 K and gradually increases to 0.70 at 110 K. The gradual change is consistent with the complex nature of the hydrogen disordering transition. Near 104 K the Raman spectra resemble more the ones of ice VI, but at 110 K and above a closer similarity with the spectra of ice XV is noted. However, the spectra are neither identical to the ice VI nor to the ice XV reference spectra. This suggests that the 1.80 GPa sample has indeed to be viewed as inhomogeneous, as also indicated by dielectric spectroscopy. Taken together it has become clear that also Raman spectroscopy provides evidence for a distinct ice polymorph more hydrogen ordered than ice XV present in an inhomogeneous sample recovered from 1.80 GPa.

### Neutron diffraction from deuterated samples

In order to learn more about the nature of the hydrogen order in the new polymorph, in particular whether or not it displays ferroelectricity and what its space group is, neutron diffraction experiments on deuterated samples would be desirable. However, in the fully deuterated samples studied for this work a second endotherm, analogous to that observed in Fig. 2, does not appear, *i.e.*, a pronounced isotope effect precludes such experiments.^3^,^14^ Specifically a DCl-doped D_2_O sample cooled at 1.4 GPa at 0.5 K min^–1^ does not show the second endotherm. This is consistent with the neutron diffraction results obtained by Salzmann *et al.*^10^: a deuterated sample prepared the way just described did not show any indications of a new polymorph but rather the signatures of ice XV. Also Komatsu *et al.* did not report any hints for the existence of a phase other than ice VI and ice XV in deuterated samples, even though the pressure range up to 1.60 GPa was investigated.^3^ For this reason the isotope effect on the ordering transition requires further study.

### Powder X-ray diffraction

For a crystallographic comparison of the new polymorph prepared in this work with conventionally prepared ice XV we used powder X-ray diffraction on H_2_O samples. Hydrogen atoms barely scatter X-rays. Therefore, different types of hydrogen ordering can be inferred merely from indirect effects such as slight changes in the oxygen network, as for instance exploited by Kamb *et al.* for the example of ice II.^29^

**Fig. 10:**
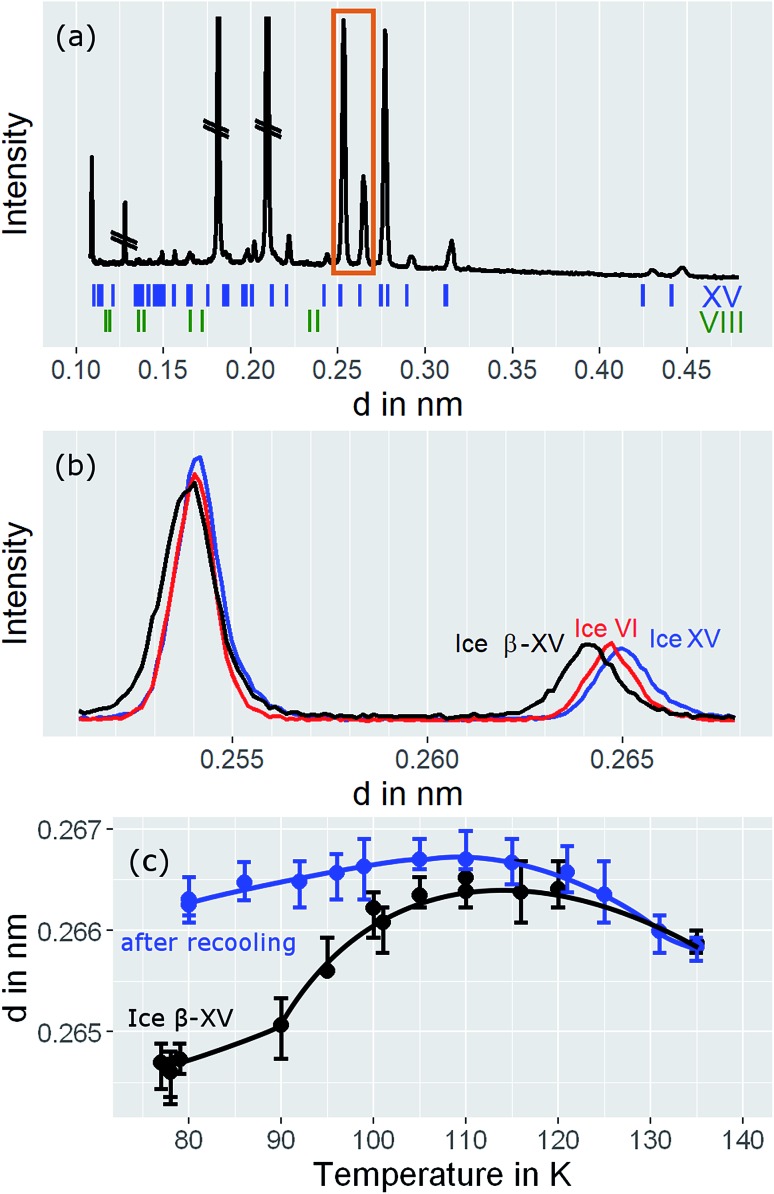
(a) Powder X-ray diffractograms of ice β-XV recorded at 20 K. Crossed out peaks stem from the sample holder. (b) Comparison of diffraction pattern for ice VI, ice XV, and ice β-XV at 80 K in the range highlighted by the orange rectangle in (a). (c) Peak positions for ice β-XV at different temperatures (black trace), and for ice β-XV after recooling from 118 K, *i.e.*, for ice XV (blue trace). The lines are a guide to the eye. Note that the diffractograms in (a) were recorded on the XPERT III, and the diffractograms for (b) and (c) on the D5000 diffractometer.

Indeed, the X-ray diffractogram in Fig. 10(a) recorded at 20 K for the 1.80 GPa sample is very similar to the known X-ray diffractograms of ice VI 30 and ice XV 10 (see ticks). This demonstrates that the oxygen networks of all three samples are almost identical. Hence, both endotherms in Fig. 2 are indeed associated with hydrogen disordering rather than with significant changes in the oxygen lattice. However, the (102) Bragg peak in ice VI near a lattice plane spacing of *d* = 0.265 nm (see Fig. 10(b), diffractogram recorded at 80 K) displays important differences among different phases. In ice XV this peak is shifted to higher *d*-spacing compared to ice VI whereas in the new polymorph it is shifted to lower *d*-spacing.^14^

### Behaviour of 1.80 GPa sample upon heating

The temperature dependence of the lattice spacing (Fig. 10(c)) for the (102) Bragg peak in ice VI for the 1.80 GPa sample (black symbols) is quite revealing. Up to 104 K there is a pronounced increase in the lattice plane spacing, which then flattens. The flattening is associated with the hydrogen disordering marked by the first endotherm in Fig. 2. Above 120 K the lattice spacings decrease, consistent with the second endotherm in Fig. 2, *i.e.*, the transition to ice VI. Other Bragg peaks, *e.g.*, the one near *d* = 0.254 nm do not show this phenomenology.^14^,^15^ This kind of U-shape in the temperature evolution of the *d* = 0.265 nm Bragg peaks was observed previously in ref. 14. In order to investigate whether this U-shape also appears for ice XV samples we have recooled the 1.80 GPa sample to form ice XV. Fig. 10(c) (blue symbols) shows that the U is much shallower for the 1.80 GPa_rec(118 K)_ sample than for the 1.80 GPa sample containing domains of the new hydrogen ordered polymorph. The steeply increasing part that is observed for the 1.80 GPa sample up to 104 K is missing for the 1.80 GPa_rec(118 K)_ sample. Instead, the data of the 1.80 GPa_rec(118 K)_ sample show overlap with those of the 1.80 GPa sample above 124 K, when both finally transform to disordered ice VI. Above 124 K, all Bragg peaks converge to the position of the ice VI Bragg peaks. However, below 124 K there is a clear discrepancy in the Bragg peak position between ice XV and the new polymorph. This rules out that the new polymorph is identical to ice XV and again demonstrates the presence of three distinct phases upon heating in the sample prepared at 1.80 GPa. All of the three distinct phases share a highly similar oxygen network. Since with ice VI and ice XV only two such phases are known, our findings imply the discovery of a novel variant of ice XV/VI.

## Discussion

It is suggested that our experimental data imply the presence of a new hydrogen ordered phase of ice VI. Here, we discuss arguments indicating that alternative interpretations can be ruled out. An important question we consider is, whether or not the new polymorph is the same as a known ice phase or a mixture of different known phases. The X-ray data clearly exclude all ice polymorphs except for ice VI and its hydrogen ordered proxies.^14^ Other oxygen atom networks, such as the ones in ice V, VIII or ice XII show completely different X-ray patterns (*cf.* the ice VIII ticks in Fig. 10(a)).^31^–^33^ Therefore, it remains to check whether the new polymorph is indeed a previously experimentally unknown phase or whether it rather stems from pure ice VI, pure ice XV or an ice VI/XV mixture. Previous experiments showed that upon heating ice VI in a calorimeter, an orientational glass transition is visible as a step in heat capacity with an onset temperature of ≈132 K, directly followed by heat release at ≈148 K, associated with the exothermic ice VI → ice I transition.^15^,^34^ For ice XV an endothermic transition to ice VI at ≈121 K, associated with hydrogen disordering, is known to precede the exotherm of the ice VI → ice I transition.^15^ For the 1.80 GPa sample studied here, both the exotherm at ≈148 K and the endotherm at ≈121 K are observed. However, a second endotherm appears additionally at ≈103 K (Fig. 2). This endotherm, which we identify as a phase transition, does not appear upon heating ice VI or ice XV. This implies that the new polymorph cannot be pure ice VI, pure ice XV or a mixture thereof.

The dielectric loss data are consistent with this conclusion. For the 1.80 GPa sample the appearance of three linear regimes in the relaxation map suggests the presence of three phases. The location of the two kinks separating the three phases is close to the location of the two endotherms in Fig. 2. At ambient pressure, the low-temperature transition is non-reversible, therefore sample 1.80 GPa_rec(114 K)_ only displays one kink upon heating, consistent with the results for the ice XV reference sample.

It is not entirely clear from our data whether or not the phase identified in this work transforms first to ice XV near 100 K and then to ice VI above 120 K. Such a two-step disordering separated by more than 20 K would be unique. We are not aware of a related two-step disordering neither for H_2_O ice nor for systems such as spin ices.^35^–^38^ An alternative interpretation is that the 1.80 GPa sample is inhomogeneous and contains the new phase mixed with ice XV domains. The broadening of the dielectric spectra observed for the 1.80 GPa sample below 105 K indeed suggests the sample to be inhomogeneous, hinting at the existence of domains with different hydrogen order. The most likely scenario is that domains from both ice XV and the new polymorph are present in the 1.80 GPa sample.

Raman spectroscopy indicates the new polymorph to be more ordered than ice XV based on more narrow and structured bands in the former than the latter sample. Analysis of the transformation behavior upon heating suggests that the new polymorph transforms neither to ice VI nor to ice XV upon heating to 103 K, but adopts a hydrogen ordering pattern different from these two phases. It needs to be settled first what the Raman spectra of ice XV at 115 K really imply before Raman spectroscopy allows one to clarify whether the new polymorph develops towards ice XV or not. The difference between the spectra of ice XV recooled from 130 K (Fig. 8, middle), recooled from 120 K (Fig. 9(b)) and ice XV without recooling noted in our work requires further investigation. At 130 K the ice VI phase is clearly reached, but it is not entirely settled whether there is a direct transformation from the new polymorph to ice VI, whether the transformation takes place *via* ice XV or whether the transformation takes place *via* states of hydrogen ordering distinct from ice VI as well as from ice XV.

## Conclusions

In summary, we found strong indications for the existence of a second hydrogen ordered phase of ice VI. The new phase revealed by our 1.80 GPa sample was carefully compared with ice XV, both in its “pristine” form obtained from cooling at 1.00 GPa as well as in its recooled form obtained at 1 bar. The phase behavior is markedly different for the various samples as consistently observed in X-ray, dielectric, calorimetric and Raman experiments. The observation of a discontinuous transition involving latent heat in Fig. 2 as well as the sudden change of activation energies indicated in the relaxation map of Fig. 7 underscore that the new variant of ordered ice VI is indeed a new phase. It remains to be determined whether it represents the ferroelectric *Cc* phase originally envisaged for ice XV which is now considered to be antiferroelectric.^6^,^10^,^11^ In principle, the crystal structure of the new polymorph might show any of the 45 possible types of dipole ordering detailed by Komatsu *et al.*^3^ Knowledge of the crystal structure based on a diffraction experiment is traditionally required to assign a unique Roman numeral to a new ice phase.^39^,^40^ Since we do not have this information at hand we suggest the second ordered variant of ice VI tentatively to be called ice β-XV and to designate it properly, namely ice XVIII, as soon as its crystal structure is published.

Based on the calorimetry and Raman results the new polymorph seems to be more ordered and hence thermodynamically favored over ice XV at temperatures below 103 K. This means that ice β-XV represents a new stable phase. Its stability range in the phase diagram is shown as the colored rectangle in Fig. 1. According to the Clapeyron relation 
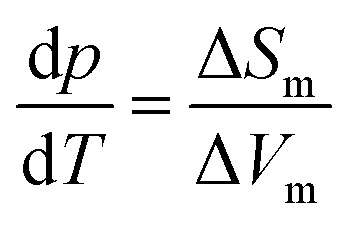
 the phase boundaries between the ice β-XV/ice II and the ice β-XV/ice VIII neighbors have to be almost vertical. This is because of the small change in entropy occurring at the phase transition between two ordered phases of differing densities. Furthermore, the densities of ice XV and ice β-XV are similar, as inferred from the X-ray experiments. Therefore, the phase boundary between these two phases needs to be almost horizontal, resulting in an almost rectangular stability domain. Thus, two triple points, each associated to three hydrogen ordered phases, are identified at *T*/*p*_II–XV–βXV_ = 103 K/0.8 GPa and *T*/*p*_VIII–XV–βXV_ = 103 K/1.5 GPa. The phase boundaries of the ices II–XV and XV–VIII are based on work of Salzmann *et al.*^10^ The location of these phase boundaries at 0.8 and 1.5 GPa is based on the intersection of the VI–XV boundary at ≈130 K with the extrapolated II–VI and VI–VIII boundaries, respectively. At 1.80 GPa ice VIII is thermodynamically more stable, but the transition from ice VI to ice VIII has a much higher barrier (it involves rearrangements of O- and H-atoms) than the transition from ice VI to ice β-XV (it involves merely a rearrangement of H-atoms). That is, at 1.80 GPa ice β-XV represents a kinetically stabilized, metastable phase below 103 K, whereas it is thermodynamically stable between 0.8 and 1.5 GPa. Ice β-XV/VIII phase coexistence cannot be observed on the time scales of the experiments done here. For this reason it is possible to observe ice XV and ice β-XV in the stability domain of ice VIII without any signs of transformation.

Future experiments to further increase our understanding of hydrogen ordering will involve D_2_O samples, an investigation of the effect of quantum tunneling on the formation of ice β-XV and neutron diffraction to possibly unravel the crystal structure. Also the states of hydrogen ordering involved in the phase transitions merit further investigation.

## Author contributions

T. G. prepared all samples and conducted X-ray diffraction and calorimetry experiments, A. T. conducted Raman experiments. L. P. and K. K. conducted dielectric experiments, T. G. and T. L. analyzed Raman, X-ray and calorimetric data, A. T. analyzed Raman data and L. P., K. K. and R. B. analyzed the dielectric data, M. E. analyzed the X-ray data, T. G. and T. L. designed the research, T. G., T. L., L. P. and R. B. wrote the manuscript.

## Conflicts of interest

The authors declare no competing financial interests. Correspondence and requests for materials should be addressed to T. L. (Email: thomas.loerting@uibk.ac.at).
